# Energy, exergy and economic (3E) analysis of flat-plate solar collector using novel environmental friendly nanofluid

**DOI:** 10.1038/s41598-023-27491-w

**Published:** 2023-01-09

**Authors:** Muhammad Amar, Naveed Akram, Ghulam Qadar Chaudhary, Salim Newaz Kazi, Manzoore Elahi M. Soudagar, Nabisab Mujawar Mubarak, Md Abul Kalam

**Affiliations:** 1 Department of Mechanical Engineering, Mirpur University of Science and Technology (MUST), Mirpur, 10250 AJK Pakistan; 2 Department of Mechanical Engineering, Faculty of Mechanical Engineering, University of Malaya, 50603 Kuala Lumpur, Malaysia; 3 Department of Mechanical Engineering and University Centre for Research & Development, Chandigarh University, Mohali, Punjab 140413 India; 4 Department of Mechanical Engineering, School of Technology, Glocal University, Delhi-Yamunotri Marg, Saharanpur, 247121 Uttar Pradesh India; 5 Department of VLSI Microelectronics, Saveetha School of Engineering, Saveetha Institute of Medical and Technical Sciences, Chennai, 602105 Tamilnadu India; 6 Petroleum and Chemical Engineering, Faculty of Engineering, Universiti Teknologi Brunei, Bandar Seri Begawan, BE1410 Brunei Darussalam; 7 School of Civil and Environmental Engineering, FEIT, University of Technology Sydney, Ultimo, NSW 2007 Australia

**Keywords:** Environmental sciences, Energy science and technology, Engineering, Materials science, Nanoscience and technology

## Abstract

The use of solar energy is one of the most prominent strategies for addressing the present energy management challenges. Solar energy is used in numerous residential sectors through flat plate solar collectors. The thermal efficiency of flat plate solar collectors is improved when conventional heat transfer fluids are replaced with nanofluids because they offer superior thermo-physical properties to conventional heat transfer fluids. Concentrated chemicals are utilized in nanofluids' conventional synthesis techniques, which produce hazardous toxic bi-products. The present research investigates the effects of novel green covalently functionalized gallic acid-treated multiwall carbon nanotubes-water nanofluid on the performance of flat plate solar collectors. GAMWCNTs are highly stable in the base fluid, according to stability analysis techniques, including ultraviolet–visible spectroscopy and zeta potential. Experimental evaluation shows that the thermo-physical properties of nanofluid are better than those of base fluid deionized water. The energy, exergy and economic analysis are performed using 0.025%, 0.065% and 0.1% weight concentrations of GAMWCNT-water at varying mass flow rates 0.010, 0.0144, 0.0188 kg/s. The introduction of GAMWCNT nanofluid enhanced the thermal performance of flat plate solar collectors in terms of energy and exergy efficiency. There is an enhancement in efficiency with the rise in heat flux, mass flow rate and weight concentration, but a decline is seen as inlet temperature increases. As per experimental findings, the highest improvement in energy efficiency is 30.88% for a 0.1% weight concentration of GAMWCNT nanofluid at 0.0188 kg/s compared to the base fluid. The collector's exergy efficiency increases with the rise in weight concentration while it decreases with an increase in flow rate. The highest exergy efficiency is achieved at 0.1% GAMWCNT concentration and 0.010 kg/s mass flow rate. GAMWCNT nanofluids have higher values for friction factor compared to the base fluid. There is a small increment in relative pumping power with increasing weight concentration of nanofluid. Performance index values of more than 1 are achieved for all GAMWCNT concentrations. When the solar thermal collector is operated at 0.0188 kg/s and 0.1% weight concentration of GAMWCNT nanofluid, the highest size reduction, 27.59%, is achieved as compared to a flat plate solar collector with water as a heat transfer fluid.

## Introduction

The world's population and energy consumption are rapidly expanding. Modern human cultures' industrialization and globalization are major causes of this increase in energy consumption. The International Energy Agency predicts that by the end of 2040, global energy consumption will grow approximately 30%^1^. Fossil fuels satisfy 86% of worldwide energy demand^2^. The world's fossil fuel reserves are rapidly depleting, and the environment is severely polluted. The challenge in the current era is to fulfill energy demands without further degrading the environment. Sustainable Development Goal 7 becomes a challenge that confront every country and affects everyone. The fundamental objective of Sustainable Development Goal 7 is to achieve energy that is economical, clean, efficient, reliable and accessible to all people. Because conventional energy resources are finite, the search for alternative energy sources has intensified worldwide. Renewable energy resources have proved that they can meet the need for clean energy^3^.

Due to its affordability and availability, solar energy is in more demand than other renewable energy resources. Solar energy can be used in a variety of ways. Solar heat may be utilized for various applications, including space heating, household hot water, cooling, and even process heating^4,5^. Solar energy collection and conversion is a key focus in this energy sector. The sun's energy can easily be harvested and converted into thermal or electrical energy. Different equipment and technologies, such as photovoltaic and solar thermal collectors, can carry out this energy conversion process. Solar collectors employ a heat-exchanging fluid to convert solar energy to thermal energy. The absorber plate of the collector captures solar energy and transfers it to the absorber fluid, increasing its internal energy, which may subsequently be used for various purposes. Flat plate solar collectors (FPSC) with no optical concentration are utilized in the 40–100 °C range of temperature. They are suitable for household applications because of their simplicity, ease of maintenance, and minimal running costs. FPSC has relatively low efficiency and output temperature. Materials, design^6^, coating on collector plate^7^, tilt angle^8^, climatic conditions^9^, and working fluid^10^ are all factors that influence the effectiveness of Flat plate solar collectors. Substituting a greater thermal conductivity fluid for pure water (which serves as the working fluid) is one of the easiest and most effective ways to improve efficiency.

Commonly used heat transfer fluids (distilled water, glycols, oils, etc.) have limited effectiveness in heat transfer systems such as solar thermal collectors due to their low thermal conductivity and heat transfer capabilities^11,12^. Solid nanoparticles can be suspended in a base fluid to achieve high thermal conductivity. Nanofluids are defined as the dispersion of nanometer-sized particles in water with a higher thermal conductivity than ordinary water^13,14^. Choi^13^ was the first to coin the phrase "nanofluids." Masuda et al.^15^ were the first to observe a significant change in the thermophysical parameters of the base liquid following nanoparticle dispersion. These 'nanofluids' can significantly improve the heat transfer performance of ordinary fluids^16^.

Numerous studies look into utilizing nanofluids in FPSC to boost collector efficiency. According to Said et al.^17^, collector efficiency was enhanced up to 76.6% by using TiO_2_ nanofluid at a 0.00833 kg/s flow rate for 0.1% wt.% fraction of nanofluid. There was no significant difference in pressure loss and pumping power values compared to the base fluid. He et al.^18^ carried out an experimental investigation to determine the impacts of copper–water (Cu-H_2_O) nanofluid on the thermal performance of FPSC while maintaining a 140 L/h mass flow rate for different nanofluid mass fractions. A two-step method was used for nanofluid preparation. As per the experiment results, there was a substantial improvement in thermal efficiency, 23.83% for 0.1% mass concentration and 25 nm size. The efficiency of the collector was decreased with the increase in nanoparticle size. Hajabdollahi et al.^19^ conducted a study for modelling and optimization of a solar network heater using flat plate collectors. Energy efficiency and cost ratio are both regarded as two objective functions. According to the findings, it is not economically feasible in the case of high collector efficiency. Every 10% increase in fuel prices causes the cost ratio to fall by 4.75%. In a study by Said et al.^20^, the thermal efficiency of a small-scale solar-driven organic Rankine cycle coupled with a flat plate solar collector was investigated about MWCNT + WO_3_/water hybrid nanofluid and MWCNT/R141b nano-refrigerant. The ORC system's thermal and exergy efficiency was experimentally investigated at various nanofluid flow rates and concentrations. It was reported that 0.5 vol% of nanofluid concentration and 3 lpm of nanofluid flow rate in the collector resulted in a considerable increase in energy and exergy efficiency by 8.52% and 6.30%, respectively.

The thermal efficiency of the FP solar collector was examined by Ahmadi et al.^21^ using graphene nanoplatelets (GNPs) based nanofluid as heat transfer fluid. Experimental results showed an 18.87% enhancement in the collector's efficiency using graphene nanofluid. Novel Ionic liquid-MXene hybrid nanofluids' thermophysical properties were examined by Said et al.^22^. With 0.5% weight of MXene nanomaterial, the thermal conductivity of 0.82 W/mK was attained. Another study investigated the energy efficiency, visualized energy, and pollution generation of a solar flat plate collector that uses a water/copper–aluminum hybrid nanofluid^23^. It was determined that hybrid nanofluid's collector energy efficiency is greater than that of other working fluids. An experimental investigation to analyze the performance of a solar-powered shell and tube heat exchanger using MWCNTs/ water-based nanofluids was performed by Said et al.^24^. At 0.3% vol., the heat transfer coefficient was improved by 31.08%. The area was reduced by 5.4% for 0.3% MWCNT/water system compared to the base fluid. Jouybari et al.^25^ experimentally examined the thermal efficiency of FPSC using SiO_2_/deionized water nanofluid. They found an 8.1% increment in thermal efficiency. The efficiency curve's slope parameter diminishes as the nanoparticle size decreases. Kiliç et al.^26^ conducted an experimental study to check the effect of using TiO_2_/water nanofluid as a working fluid on the thermal performance of FPSC. Triton X-100 surfactant was also added during nanofluid preparation to increase the stability of the nanofluid. It was found that 48.67% maximum instantaneous efficiency was achieved. Stalin et al.^22^ conducted an experimental and theoretical study to analyze the effectiveness of liquid flat plate collectors utilizing CeO_2_-based nanofluid. Compared to base fluid water, a solar collector with cerium dioxide (CeO_2_/H_2_O) nanofluid achieved 78.2% thermal efficiency, which was 21.5% more than water. However, some studies also indicate a reduction in collector efficiency using alumina-based nanofluid^27^. The nanoparticle deposition wall formation noted a 5.5% reduction in efficiency. This deposition layer created extra thermal resistance to heat transfer, and ultimately, thermal efficiency was decreased. Arora et al.^28^ studied flat plate solar collector performance using an innovative absorber tube, i.e., Marquise Shaped and Al_2_O_3_/water nanofluid. Experimental findings illustrated that at a mass flow rate of 3lpm, collector efficiency with and without nanofluid is 83.17% and 59.72%, respectively. Another study was carried out by Akram et al.^29^ to investigate the performance of FPSC. The covalent functionalization method was adopted to synthesize green graphene-based nanofluids. There was a significant increment in the colloidal stability of nanofluid. Experimental results showed that thermal efficiency was enhanced by 18.2% by utilizing nanofluids compared to water. Choudhary et al.^30^ performed an experimental study to check the thermal behavior of the collector by using ZnO/water nanofluid. The noncovalent functionalization method was adopted to prepare the nanofluid. It was found that with time, the nanofluid becomes inefficient due to sedimentation, aided by higher particle size. The percentage enhancement in efficiency was 19.9% when compared to the base fluid. Moravej et al.^31^ utilized rutile TiO_2_/water nanofluid for the performance investigation of symmetric FPSC. Nanofluid was synthesized by a non-covalent functionalization method without using a surfactant. The use of TiO_2_-water nanofluid significantly boosted thermal efficiency. Another research was carried out by Sarsam et al.^32^ to analyze the thermal behavior of the collector by employing GNPs based nanofluid. They used the conventional/ covalent functionalization method to functionalize GNPs with triethanolamine (TEA). Although a considerable enhancement in collector efficiency was noted, strong chemicals were utilized in covalent functionalization, which produces hazardous toxic bi-products. Akram et al.^33^ used carbon and metal-based nanofluids as working fluids to analyze the thermal efficiency of FPSC. Carbon nanoplates were covalently functionalized in this study, and metal oxides were non-covalently functionalized using a surfactant. Results indicated 60 days of stability for carbon-based nanofluid and 30 days for metal-based nanofluid. The percentage enhancement in efficiency was 17.45% for carbon-based nanofluid compared to water. Kumar et al.^34^ reported that GGNPs with 0.1 wt% and 1.5 lpm flow rate resulted in a 24.09% increase in LFPSC efficiency over distilled water. Covalent functionalization of graphene nanoplatelets with gallic acid was done. Relative pumping power slightly increased with increasing GGNP concentration.

Although nanofluids based on carbon nanoparticles provide a high heat transfer rate, carbon-based nanomaterials have low colloidal stability in base fluid due to their hydrophobic nature. Therefore, it is crucial to modify the surface of carbon-based nanoparticles to enhance their colloidal stability. Surface modification can be done using either covalent or non-covalent functionalization methods. Surfactants are required for non-covalent functionalization, which has unwanted consequences such as foam generation, corrosion, and many others. Therefore, to achieve long-term dispersible stability covalent functionalization method is preferred. Thermo-physical characteristics of working fluids are also enhanced in covalent functionalization^32,35^.

Furthermore, concentrated chemicals are utilized in covalent functionalization, producing hazardous toxic bi-products^35,36^. As a necessity, the use of environmentally acceptable ingredients for synthesizing nanoparticles, particularly carbon-based nanoparticles, is essential.

Gallic acid (GA), a polyphenol antioxidant, is present in many fruits and vegetables, including grapes and tea^37^. The pharmaceutical sector uses gallic acid extensively. Due to eco friendly properties, GA can be used to covalently functionalize multiwall carbon nanotubes, converting their hydrophobic surface to hydrophilic and enhancing their stability in the base fluid.

According to the available literature, studies focused on using environmentally friendly, stable, covalently functionalized nanofluids to evaluate the thermal performance of FPSCs are not discovered. In the present study, a green, long-term stable, covalently functionalized gallic acid-treated multiwall carbon nanotubes-water nanofluid is used as heat transfer fluid to evaluate the energy and exergy efficiency of a flat plate solar collector. Economic analysis of FPSC utilizing green synthesized GAMWCNT-water nanofluid is also carried out. The effect of outlet temperature on collector efficiency, friction factor (f), pumping power, collector size reduction, and cost reductions are assessed. The experiments are carried out with three different weight concentrations of GAMWCNT-H_2_O nanofluid, 0.025%, 0.065%, and 0.1%, at varying mass flow rates of 0.010, 0.0144, and 0.0188 kg/s, while maintaining heat flux intensities of 600, 800, and 1000 W/m^2^ and temperatures at the inlet between 303 and 323 K.

## Materials and methods

The following three topics are covered in depth in this section:

1. Eco-friendly covalent functionalized GAMWCNTs nanofluids synthesis technique
2. FPSC Experimental test rig. for testing thermal performance
3. Testing method for conduction of experiments utilizing Eco-friendly nanofluid

### Synthesis of Green GAMWCNTs nanofluid

**Figure 1:**
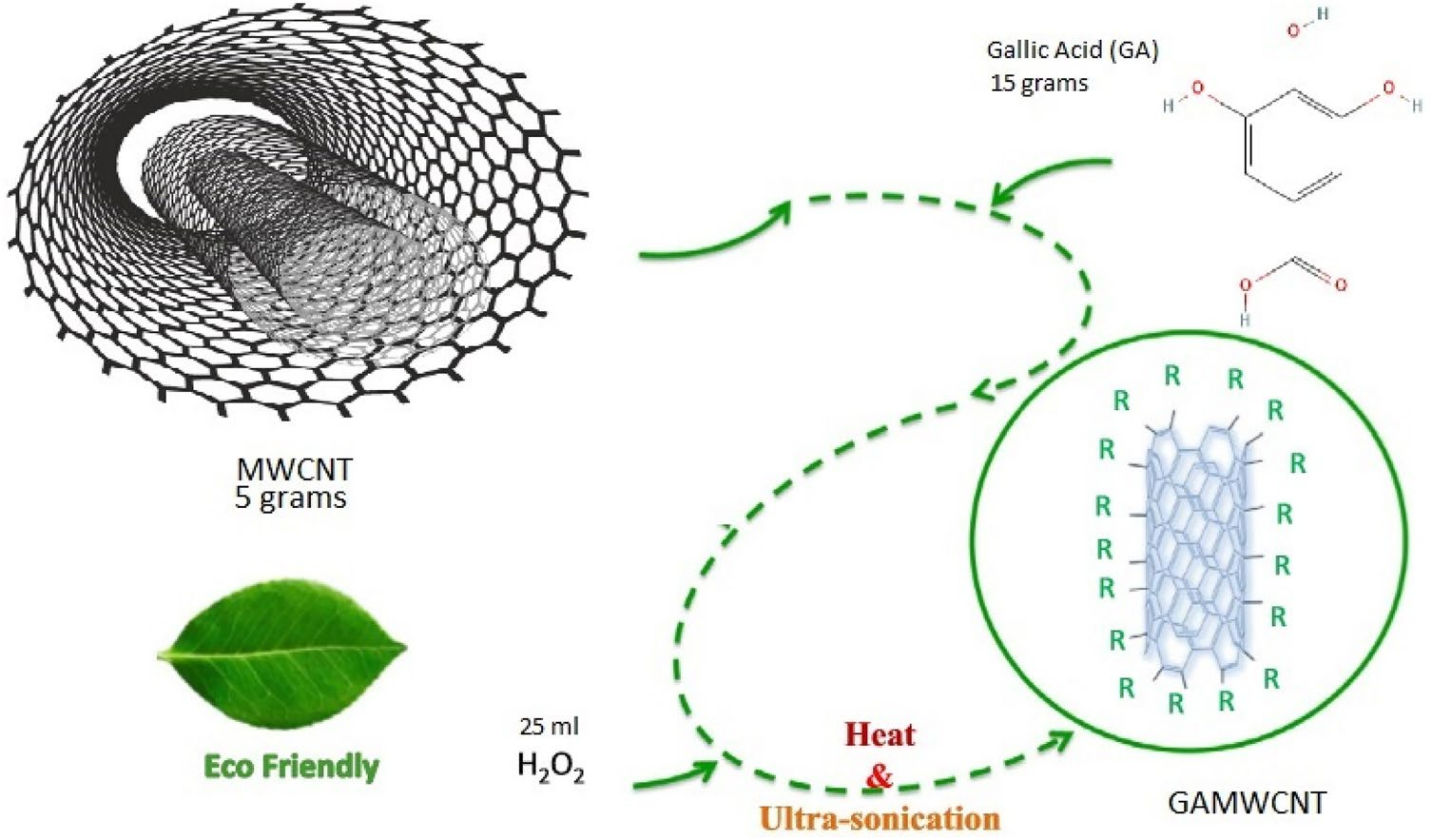
Graphical presentation of the synthesis process.

Natural phenolic extract 3,4,5 trihydroxy benzoic acid, also known as gallic acid (GA), was used to covalently functionalize multiwall carbon nanotubes (diameter: < 8 nm, purity: > 95%, SSA: > 500 m^2^/g). A two-step method, as suggested by Akram et al.^38^ was introduced for preparation of green gallic acid treated multiwall carbon nanotubes nanofluid. 5 g of immaculate multi-wall carbon nanotubes (Nanostructured & Amorphous Materials Inc.), and 15 g of gallic acid were immersed into a beaker filled with 1000 ml distilled water and then stirred for almost 1/4 h until the mixture turned homogeneous. During the sonication time, 25 ml of H_2_O_2_ (Brand-sigma-Aldrich) was injected dropwise into the mixture. The resulting mixture was ultra-sonicated for 1/3 h. The mixture was then refluxed for 14 h at 80 °C. The centrifugation of GAMWCNs colloid was carried out at 14,000 rpm and rinsed multiple times with distilled water to eliminate residual particles until the pH reached 7. Afterward, the synthesized specimen was dried at 60 °C in an oven for a day. Finally, gallic acid-treated multi-wall carbon nanotubes -water nano-fluid was synthesized by dispersing 0.025, 0.065, and 0.1 wt.% covalently functionalized MWCNTs nanoparticles in water for 10 min via ultra-sonication. The GAMWCNTs were found to be well-dispersed in the base fluid. A schematic diagram of the synthesis of GAMWCNT is shown in Fig. 1.

### Experimental setup

**Figure 2:**
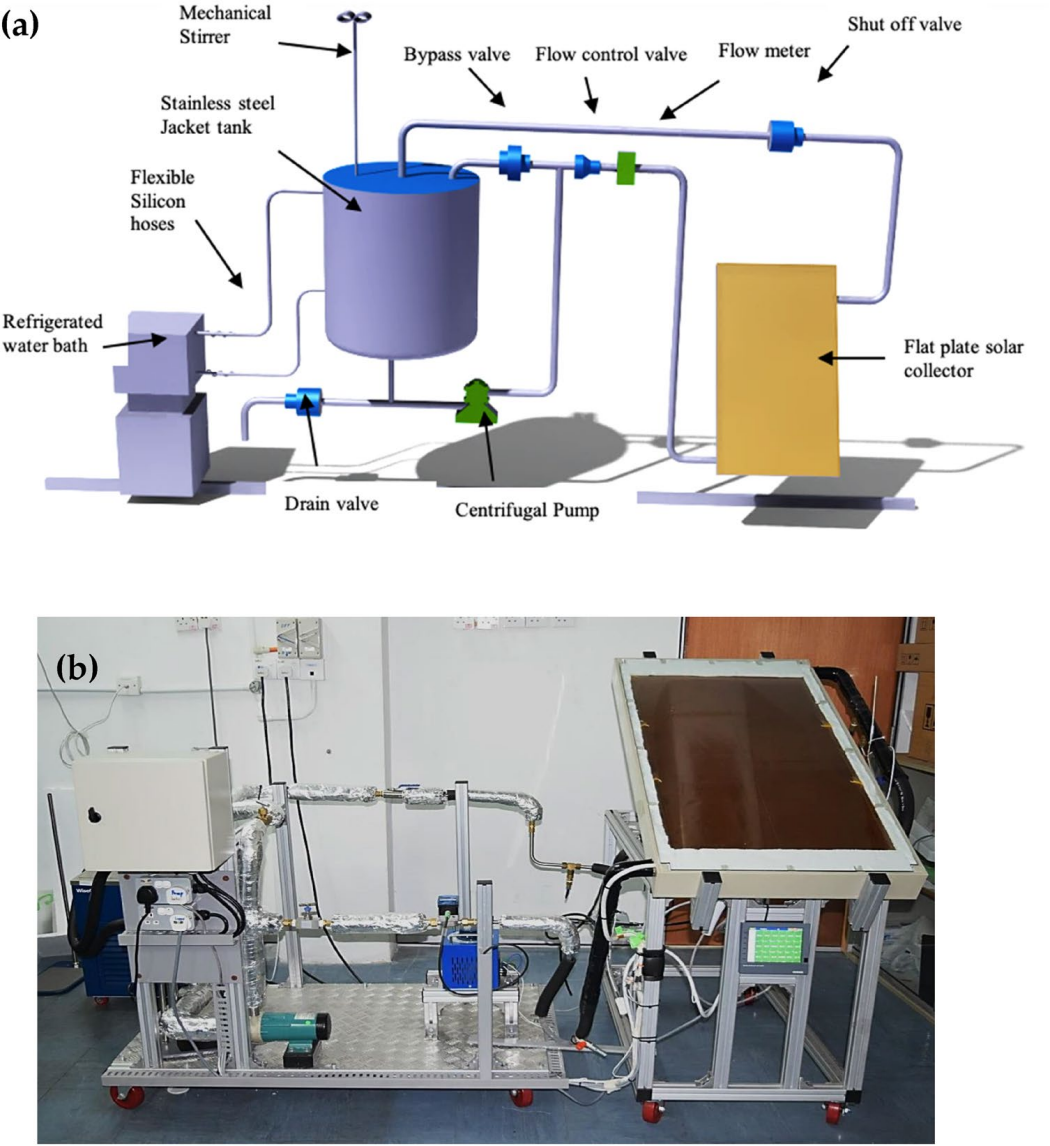
(**a**) Schematic view of experimental setup. (**b**) Pictorial view of the experimental setup.

**Figure 3:**
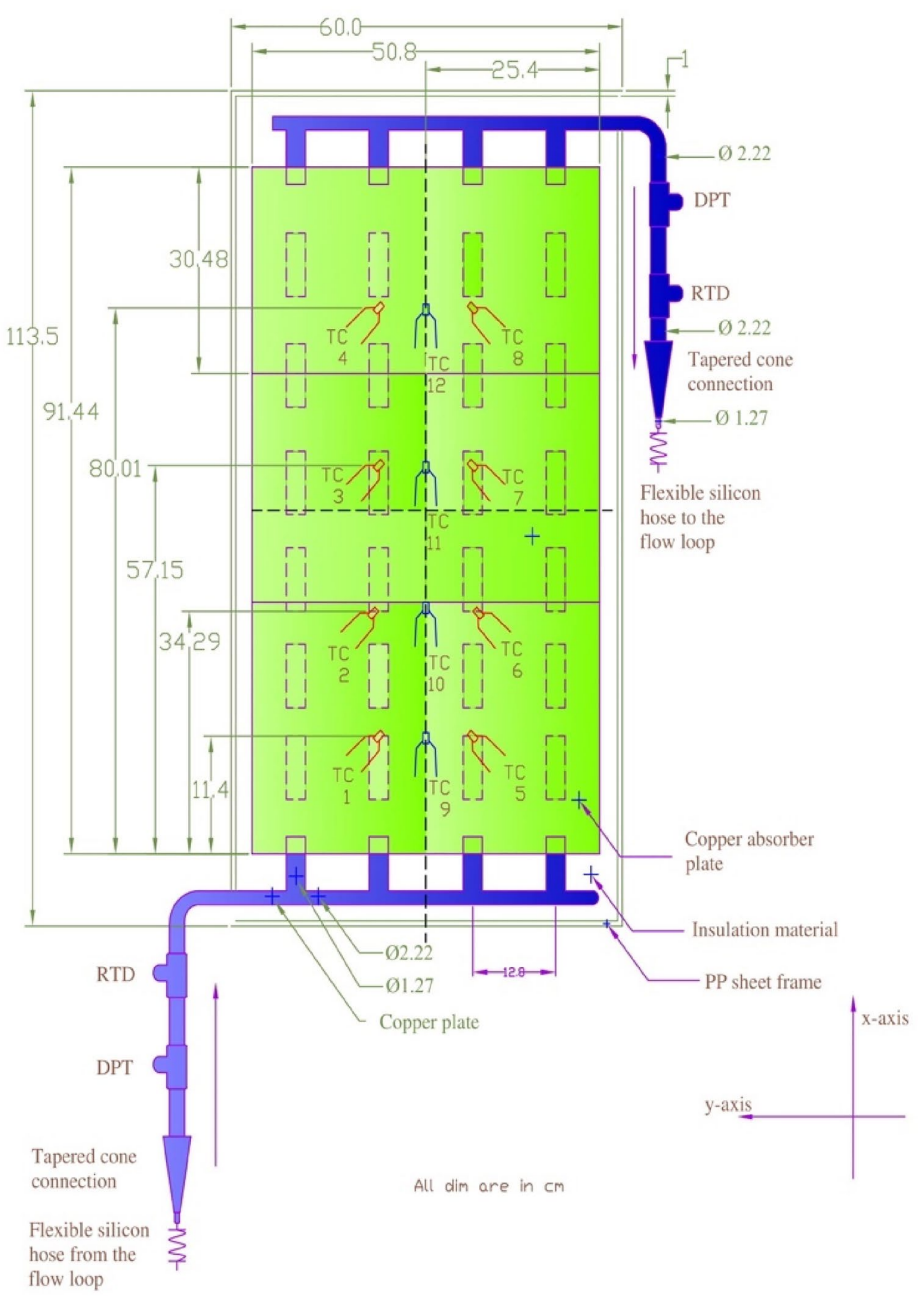
Detail description of FPSC along with thermocouple positions.

Experiments were carried out in a Laboratory at the Mechanical Engineering Department of the University of Malaya in Malaysia (indoor testing) to analyze the thermal performance of flat plate solar thermal collectors. Table 1 lists the detailed specifications of the FPSC utilized in this investigation. Figure 2a shows a schematic representation of the test rig arrangement, while Fig. 2b represents the pictorial view of the experimental setup. The test rig includes several key parts such as a flow loop, control devices, data logger, refrigerated water bath circulator (cooling medium) and FPSC. The inside view of FPSC and detail of thermocouples installation on the riser tubes are shown in Fig. 3. A centrifugal electric pump was used to circulate the working fluid in the forced convection system. A self-adhesive T-type thermocouple measured ambient temperatures, and a flexible adhesive heater fixed at the collector surface was used as a source of constant heat flux akin to solar irradiance. A refrigerated water bath circulator with an insulated jacketed tank was employed to control the nanofluid temperature at the collector's inlet. A stainless-steel storage tank with a capacity of 8 l was utilized as storage of working fluid(nanofluid), and to measure mass flow rate, a digital flow meter was installed. A needle valve was inserted well before the flow meter to control the mass flow rate during the testing, and calibrated PT-100 resistance temperature detectors (RTDs) were used to monitor the solar collector's intake and outlet temperatures.

**Table 1:** Specification of the test setup.

Sr.No	Specification	Value	Unit
1	Length	113.5	cm
2	Width	60	cm
3	Depth	9	cm
4	Collector area	0.6810	m^2^
5	Absorption area of the collector	0.4645	m^2^
6	The thickness of the glass cover	0.5	cm
7	The outer diameter of the collector header tube (Do)	2.22	cm
8	The inner diameter of the collector header tube (Di)	2.09	cm
9	The outer diameter of the collector riser tube (do)	1.27	cm
10	The inner diameter of the collector riser tube (di)	1.16	cm
11	Spacing between tubes	12.8	cm
12	Glass cover emissivity	0.88	–
13	Absorption coefficient	0.95	–
14	Insulation on back	5	cm
15	Insulation on sides	3	cm
16	Insulation thermal conductivity	0.04	w/mK
17	Tilt angle	30	degree
18	Number of the riser tube	4	–

In four different positions along the x-axis, the exterior wall temperatures of the two middle riser tubes (TW) and the central line temperature of the absorber plate (AP) were determined, as seen in Fig. 3. It can be observed that the positions of thermocouples are 11.4, 34.29, 57.15, and 80.1 cm from the reference position of an absorber plate in the direction of the x-axis. It may call the dimensionless axial distance (x/d). The value of TW was taken by averaging two riser tubes at each position. The outlet, absorber plate (AP), tube wall (TW), and ambient temperature and pressure drop for each test run were recorded at steady-state conditions and heat flux intensities of 600, 800, and 1000 W/m^2^ as suggested by the ASHRAE Standard 93-2003 for indoor testing^39^.

### Testing procedure

A flat plate solar collector's thermal performance was tested indoors per ASHRAE standard 93-2003^39^. All tests were carried out in steady-state conditions with various heat flux rates equivalent to solar radiations to determine the FPSC's thermal efficiency.

Useful heat gain under steady-state conditions is calculated using Eq. (1)^40^

1 $${Q}_{u}={{\dot{m}}}{C}_{p} ({T}_{o}-{T}_{i})$$

The thermal efficiency of the FP solar collector is computed using Eq. (2)^32,41^

2 $$\eta_{T} = { }{\raise0.7ex\hbox{${Q_{u} }$} \!\mathord{\left/ {\vphantom {{Q_{u} } {A_{c} .G_{T} }}}\right.\kern-0pt} \!\lower0.7ex\hbox{${A_{c} .G_{T} }$}} = { }\dot{m}C_{p} \left( {T_{o} - T_{i} } \right)/A_{c} .G_{T} = { }F_{R} .\left( {\tau \alpha } \right) - F_{R} U_{L} \left( {\frac{{T_{o} - T_{i} }}{{G_{T} }}} \right)$$

Equation (3) is used for finding the heat removal factor^41^

3 $${F}_{R}= = \dot{m}{C}_{p}\left({T}_{o}-{T}_{i}\right)/{A}_{c}\left[{G}_{T} \left(\tau \alpha \right)- {U}_{L }\left({T}_{O}- {T}_{i}\right)\right]$$

Exergy loss can be calculated by^41^

4 $${\dot{E}}_{dest}= {\dot{E}}_{heat}- {\dot{E}}_{\dot{{m}_{in}}}- {\dot{E}}_{\dot{{m}_{out}}}- {\dot{E}}_{work}$$

5 $$\sum \left(1-\frac{{T}_{a}}{{T}_{surr}}\right) \dot{{Q}_{s}}- \dot{W+}\sum \dot{{m}_{in} . }{\psi }_{in}- \sum \dot{{m}_{out} . }{\psi }_{out}= {\dot{E}}_{dest}$$

6 $$\sum \left( {1 - \frac{{T_{a} }}{{T_{{surr}} }}} \right){\text{~}}\dot{Q}_{s} - {\text{~}}\dot{m}\Delta h - {\text{~}}T_{a} \Delta s = {\text{~}}\dot{E}_{{dest}}$$

Absorbed energy ${\dot{Q}}_{s}$ is given by^34^

7 $$\dot{{Q}_{s}}= {G}_{t}\left(\tau \alpha \right){A}_{c}$$

Enthalpy changes of GAMWCNT nanofluid are calculated as^34^

8 $$\Delta h= \left({h}_{out}- {h}_{in}\right)= {C}_{p}\Delta T$$

And for entropy changes following Eq. (9) is used^34^

9 $$\Delta s = ~\left( {s_{{out}} - ~s_{{in}} } \right) = ~\dot{m}C_{p} \ln ~\left( {{\raise0.7ex\hbox{${T_{{out}} }$} \!\mathord{\left/ {\vphantom {{T_{{out}} } {T_{{in}} }}}\right.\kern-\nulldelimiterspace} \!\lower0.7ex\hbox{${T_{{in}} }$}}} \right) - RIn\frac{{P_{{out}} }}{{P_{{in}} }}$$

Putting values of terms from Eqs. (7–9) in Eq. (6),

10 $${\dot{E}}_{dest}= \left(1-\frac{{T}_{a}}{{T}_{s}}\right){G}_{t}\left(\tau \alpha \right){A}_{c}- \dot{m}{C}_{p}\Delta T+ \dot{m.}{T}_{a}\left[{C}_{p}ln\left(\frac{{T}_{out}}{{T}_{in}}\right)-R.{T}_{a}ln\left(\frac{{P}_{out}}{{P}_{in}}\right)\right]$$

The rate of exergy loss and exergy efficiency is found by Eq. (11) and Eq. (12)^34^

11 $${\dot{E}}_{dest}= \dot{{S}_{gen}} .{T}_{a}$$

12 $${\eta }_{e}=1- \frac{\dot{{S}_{gen}} .{T}_{a}}{\left[1- \frac{{T}_{a}}{{T}_{surr}}\right]\dot{{Q}_{s}}}$$

Experimental friction factor as evaluated from pressure drop values given by^34,42^

13 $$f= \frac{\Delta {P}_{exp}}{\left(\frac{L}{{D}_{i}}\right)\left(\frac{\rho {v}^{2}}{2}\right)}$$

Equations (14) and (15) are used for finding the Reynolds number and fluid flow velocity^34^

14 $$Re= \frac{4\dot{m}}{\pi {D}_{i}\mu }$$

15 $$V= \frac{\dot{m}}{\pi \rho {D}_{i}^{2}/4}$$

The following correlations can be used for finding theoretical friction factors^43,44^

16 $$f = { }\left[ {0.79\left( {\ln Re} \right) - 1.64} \right]^{ - 2} {\text{for}} {\text{Re range }}\left( {{23}00 \, {-}{ 1}0{5}} \right)$$

17 $$f = 0.3164Re^{{ - \left( \frac{1}{4} \right)}} {\text{for Re range }}\left( {{3}000 - {1}0{5}} \right)$$

Pumping power is evaluated by^11^

18 $$Pumping Power= \left(\frac{\dot{m}}{\rho }\right)\Delta {P}_{exp}$$

Equation (19) is used for finding relative pumping power^45^

19 $$\frac{{Z}_{nf}}{{Z}_{bf}}= {\left(\frac{{\rho }_{bf}}{{\rho }_{nf}}\right)}^{2}{\left(\frac{{\mu }_{nf}}{{\mu }_{bf}}\right)}^{3}$$

${Z}_{nf}$ and ${Z}_{bf}$ are pumping power of CMWCNT base nanofluid and base fluid, respectively.

The Performance Index (PI) is used to assess the feasibility and efficacy of GAMWCNT nanofluids in FPSCs, which is given by^46^

20 $$PI = \frac{{Z_{{\eta T}} }}{{Z_{{\Delta P}} }} = \frac{{\left( \eta \right)_{{nf}} /\left( \eta \right)_{{bf}} }}{{\left( {\Delta P_{{\exp }} } \right)_{{nf}} /\left( {\Delta P_{{\exp }} } \right)_{{bf}} }}$$

The size reduction of Flat plate solar collector is given as^47,48^

21 $${A}_{RC}= \frac{\dot{m}{C}_{p}\left({T}_{o}-{T}_{i}\right)}{{G}_{T}{\eta }_{T}}$$

### Uncertainty analysis

There are minimal flaws and inaccuracies in the data obtained. Errors cannot be avoided in any experimentation that causes uncertainty in the outcomes. An uncertainty analysis was performed to evaluate the recorded experimental values' accuracy^49^. The efficiency of FPSC will be explained proportionately in this approach, as shown by Eqs. (22) and (23)

22 $${\eta }_{c}\propto \frac{Useful\,\, energy\,\, collected}{Flux\,\, of\,\, heat\,\, available\,\, at \,\,surface} \propto \frac{\rho \dot{Q}{C}_{p }({T}_{o}- {T}_{i})}{{I}_{T}}$$

R = R (x_1_, x_2_, …x_n_) if R is the output of the provided function that is proportional to the independent parameters x_1_, x_2_,…x_n_. Equation (23), proposed by Moffat^61^ and Holman^62^, can be used to measure the uncertainty (δR).

23 $$\delta R= {\left\{\sum_{i=1}^{n}{\left(\frac{\partial R}{{\partial x}_{i}}{\delta x}_{i}\right)}^{2}\right\}}^{1/2}$$

The uncertainty in the experiments data of this investigation is calculated using Eq. (24). which is based on Eq. (23)

24 $$\frac{{\delta \eta }_{c}}{{\eta }_{c}}= {\left\{{\left(\frac{\delta \rho }{\rho }\right)}^{2}+ {\left(\frac{\delta \dot{m}}{\dot{m}}\right)}^{2}+ {\left(\frac{{\delta I}_{T}}{{I}_{T}}\right)}^{2}+ {\left(\frac{{\delta C}_{P}}{{C}_{P}}\right)}^{2}+ {\left(\frac{\delta V}{V}\right)}^{2}+ {\left(\frac{\delta I}{I}\right)}^{2}+ {\left(\frac{\delta \Delta T}{\Delta T}\right)}^{2}+ {\left(\frac{\delta K}{K}\right)}^{2} \right\}}^{1/2}$$

2.62%, 1.4%, 0.70%, 2%, 2.2%, 1.9%, 0.45%, 0.45% and 0.8% are uncertainty values of specific heat, density, voltage, current, solar irradiance, thermal conductivity, inlet temperature, outlet temperature and mass flow rate respectively. The uncertainty value for the efficiency of the collector is 3.90%.

## Results and discussion

### Characterization

This section discusses various techniques used to characterize materials, including FTIR, Raman spectroscopy, TEM and Zeta potential and UV/VIS spectroscopy.

**Figure 4:**
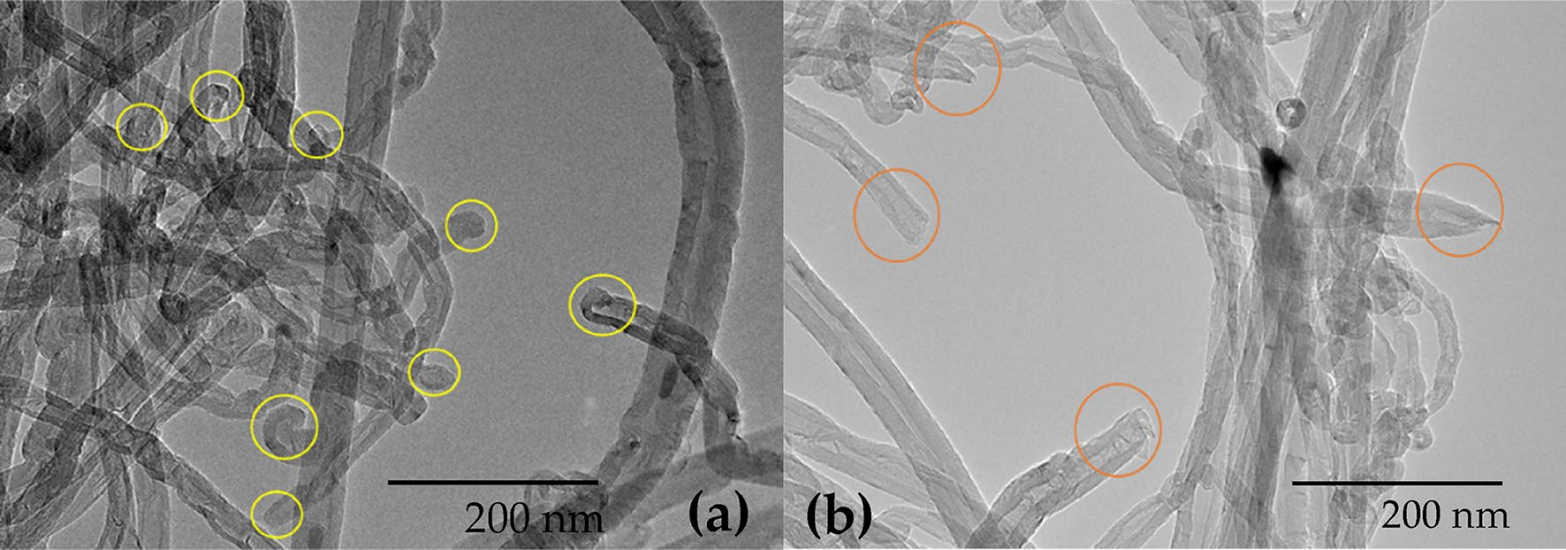
(**a**, **b**) TEM images of pristine MWCNTs and GAMWCNTs (reuse with permission of elsevier).

In the FTIR technique, compared to pure MWCNTs, the GAMWCNT sample shows a strong indication of the existence of hydroxyl (O–H) groups. The sharp and wide peaks at 3446–3750 cm^−1^ are linked with the O–H stretching vibrations at the primary structure of both MWCNTs and GAMWCNTs with various intensities due to the interaction between the MWCNTs and hydroxyl (O–H) groups of gallic acid (GA) and hydrogen peroxide (H_2_O_2_). The GA is effectively linked to the pure MWCNTs by the free-radical grafting process, as per the FTIR spectrum. Raman spectroscopy is a prominent method for determining the chemical functionalization of carbon-based materials. According to this method, both immaculate MWCNTs and GAMWCNTs feature D and G bands at wavenumbers of ~ 1350 and 1590 cm^−1^, respectively. A technique known as TEM was employed to verify the success of covalent functionalization on MWCNTs. According to TEM, the surface of MWCNTs has been successfully modified to meet the requirements, as shown in Fig. 4. Another technique, Zeta potential, is used for analyzing the stability of nanoparticles in the base fluid. As per the Zeta potential test, for a pH range of 2.70–9.56, the GAMWCNTs display strong minus values ranging from − 16 to − 52.4 mV, which are far from the point of isoelectric. The GAMWCNTs exhibit a significant electrostatic repulsion force in the pH range of 3.10–9.56, which inhibits the MWCNTs from aggregating due to noncovalent interactions. The stability of the nanofluid was also confirmed by UV/VIS spectroscopy. The absorbance reading will rise as the amount of dispersed GAMWCNTs increases, and the relative concentration of GAMWCNTs remains stable till 60 days^38^.

### Thermophysical properties of GAMWCNTs aqueous suspensions

The Thermophysical properties of GAMWCNTs and values for various concentrations are presented in Table 2.

**Table 2:** Thermophysical properties of water and GAMWCNT—water nanofluid.

Viscosity (mPa s)		Fluid Temperature (K)						
		293	297	303	308	313	318	323
DI Water	0 wt%	1.04	0.925	0.829	0.753	0.683	0.623	0.575
GAMWCNTS	0.025 wt%	1.059	0.941	0.838	0.761	0.691	0.633	0.581
GAMWCNTS	0.065 wt%	1.096	0.965	0.865	0.782	0.713	0.649	0.599
GAMWCNTS	0.1 wt%	1.116	0.991	0.879	0.797	0.727	0.664	0.61

Thermal conductivity (W/m K)								
Nanoparticle concentration in weight percentage		Fluid temperature (K)						
		293	297	303	308	313	318	323
DI Water	0 wt%	0.592	0.601	0.611	0.619	0.627	0.637	0.648
GAMWCNTS	0.025 wt%	0.608	0.621	0.63	0.645	0.659	0.669	0.685
GAMWCNTS	0.065 wt%	0.642	0.653	0.674	0.692	0.707	0.727	0.749
GAMWCNTS	0.1 wt%	0.657	0.687	0.699	0.735	0.757	0.774	0.796

Specific heat capacity (J/kg K)								
Nanoparticle concentration in weight percentage		Fluid temperature (K)						
		293	297	303	308	313	318	323
DI Water	0 wt%	4139	4141	4142	4144	4146	4149	4150
GAMWCNTS	0.025 wt%	4124	4126	4128	4128	4131	4134	4136
GAMWCNTS	0.065 wt%	4097	4099	4103	4105	4107	4110	4111
GAMWCNTS	0.1 wt%	4085	4085	4087	4088	4090	4090	4091

Density (kg/m^3^)								
Nanoparticle concentration in weight percentage		Fluid temperature (K)						
		293	297	303	308	313	318	323
DI Water	0 wt%	998.05	996.9	995.61	993.95	992.04	989.97	987.72
GAMWCNTS	0.025 wt%	998.1	997	995.6	994.05	992.1	990.1	988.05
GAMWCNTS	0.065 wt%	998.4	997.25	995.85	994.35	992.35	990.45	988.25
GAMWCNTS	0.1 wt%	998.5	997.35	995.95	994.45	992.5	990.60	988.70

An approximately 5% accurate KD2 Pro (Decagon Geräte, Inc., USA) Thermal Properties analyzer was utilized to measure the thermal conductivity of the nanofluids synthesized in this study. KS-1 prob, with a diameter of 1.3 mm and length of 60 mm used as a needle sensor, and its working principle is based on the transient hot-wire method. With less than 1% uncertainty, the recorded thermal conductivity for base fluid (DW) displays good compatibility with NIST data^50^. Compared to deionized water (DW), GAMWCNT-H_2_O nanofluids have a significantly higher thermal conductivity, as shown in Table 2, and the temperature of the working fluid and concentration of nanoparticles increases the thermal conductivity. The Brownian motion of nanoparticles in a fluid is the principal factor underpinning the increased thermal conductivity of the GAMWCNT nano-fluid, which rises with the increase in temperature. With a rise in temperature, the random mobility of nanoparticles in fluid increases. Therefore, thermal energy is transported very rapidly through the fluid. Table 2 shows that the maximum increase in thermal conductivity is 22.83% at 323 K for 0.1% weight concentration.

In this investigation, the viscosity of nanofluids was measured using an Anton Paar rotating rheometer (Anton Paar GmbH, Physica MCR 301). Shear rates ranging from 20 to 200 1/s were used for testing at various temperatures. The viscosity of the GAMWCNTs nano-fluid is greater than that of Deionized water (DW), as seen in the Table 2, while the difference is not significant. Furthermore, the effective viscosity of the GAMWCNT reduces as the temperature of the working fluid rises, which is almost equivalent to that of Deionized water (DW). Weakened intermolecular forces between the particles of the nanofluid could be the cause of this occurrence^51–53^. It can be seen that the addition of low nano-particles GAMWCNT concentration results in a small increase in viscosity value, which is beneficial because higher viscosity values diminish the effects of increased thermal conductivity of fluid owing to enhanced pumping power of heat transfer systems^54^.

Another significant thermophysical property is the specific heat capacity. Differential scanning calorimetry (DSC-Q2000, TA Instruments) was used to measure the specific heat of nanofluid produced at various weight concentrations and temperatures. Table 2 displays the specific heat capacity values recorded at various weight concentrations of GAMWCNT nanofluids and fluid temperatures. The values of specific heat capacity for deionized water (DW) are also presented for comparison. The specific heat capacity of nano-fluid based on GAMWCNT reduces as the weight concentration of nano-particles increases; when compared to base fluid deionized water, the drop in value of Cp was 0.33–1.42%, which is just a little decline. On the other hand, specific heat capacity increases with the rise in temperature of the nano-fluid.

The density of the GAMWCNT nanofluid and deionized water (DW) at various fluid temperatures and nanoparticle concentrations was also evaluated, and the findings are given in Table 2. The density of nanofluids was measured using a density meter Mettler Toledo (DM40). Due to the thermal expansion of the liquid, the density of the GAMWCNT nano-fluid and DW reduce a little as the temperature rises. It is observed that when the temperature is raised from 293 to 323 K, the density of the GAMWCNTs reduces by 0.9% for a weight fraction of 0.1 wt%. Furthermore, a linear correlation between nanoparticle concentration and density is seen, i.e., density increases with nanoparticle loading.

### Analysis of thermal efficiency

**Figure 5:**
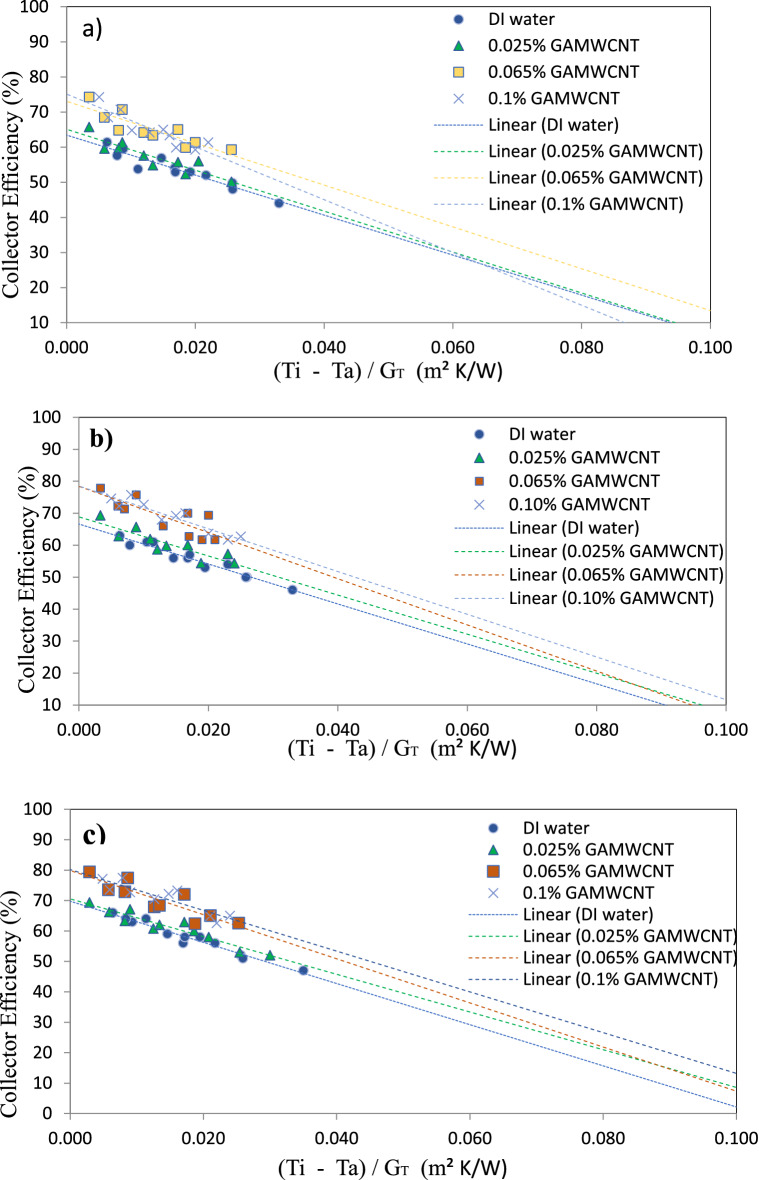
Variation in collector efficiency with $\frac{\left({T}_{i}-{T}_{a}\right)}{{G}_{T}}$ at various flow rate (**a**) 0.010 kg/s, (**b**) 0.0144 kg/s, (**c**) 0.0188 kg/s.

Figure 5 shows the variation in thermal efficiency of a flat plate solar collector for different mass flow rates and weight concentrations of GAMWCNTs nano-fluid. A drop in heat removal factor (F_R_U_L_) and rise in heat absorbed factor F_R_ (τα) are seen for the rise in mass flow rate ($\dot{m)}$. Table 3 lists the values of heat absorbed and heat removal factors for GAMWCNTs at various flow rates and weight concentrations, and these values are compared with deionized water. It can be observed that the value of F_R_ (τα) goes up with mass flow rate and is greater for GAMWCNTs nanofluid than deionized water. Increasing heat absorbed values and thermal conductivity of GAMWCNTs nano-fluid contribute to enhanced convective coefficient (h) values. As a result, the efficiency of the solar collector is seen with the increase in mass flow rate from 0.010 to 0.0188 kg/s for each GAMWCNTs weight fraction. It can be observed that at 0.1 wt.% GAMWCNTs and 0.0188 kg/s mass flow rate in comparison to base fluid deionized water, the maximum enhancement in thermal efficiency of FPSC is 30.881%. Additionally, it has been found that an improvement in LFPSC efficiency is attained with increasing weight fractions of GAMWCNTs. This is mostly because the system can absorb more energy.

**Table 3:** ${F}_{R}\left(\tau \alpha \right)$ and ${F}_{R}{U}_{L}$ factors for GAMWCNT—water nanofluid.

Mass flow rate (kg/s)	Weight concentration	${F}_{R}\left(\tau \alpha \right)$	${F}_{R}{U}_{L}$	R^2^
	(wt. %)			
0.01	0.025	0.637	5.9485	0.9752
	0.065	0.665	5.9794	0.9895
	0.1	0.6943	6.0262	0.9758
	DI Water	0.5468	5.6181	0.9821
0.0144	0.025	0.686	5.9306	0.9925
	0.065	0.7231	5.985	0.9826
	0.1	0.75	5.9992	0.9731
	DI Water	0.5878	5.5893	0.9869
0.0188	0.025	0.7235	5.925	0.9632
	0.065	0.765	5.9538	0.9784
	0.1	0.792	5.9725	0.9861
	DI water	0.6184	5.5802	0.9823

**Figure 6:**
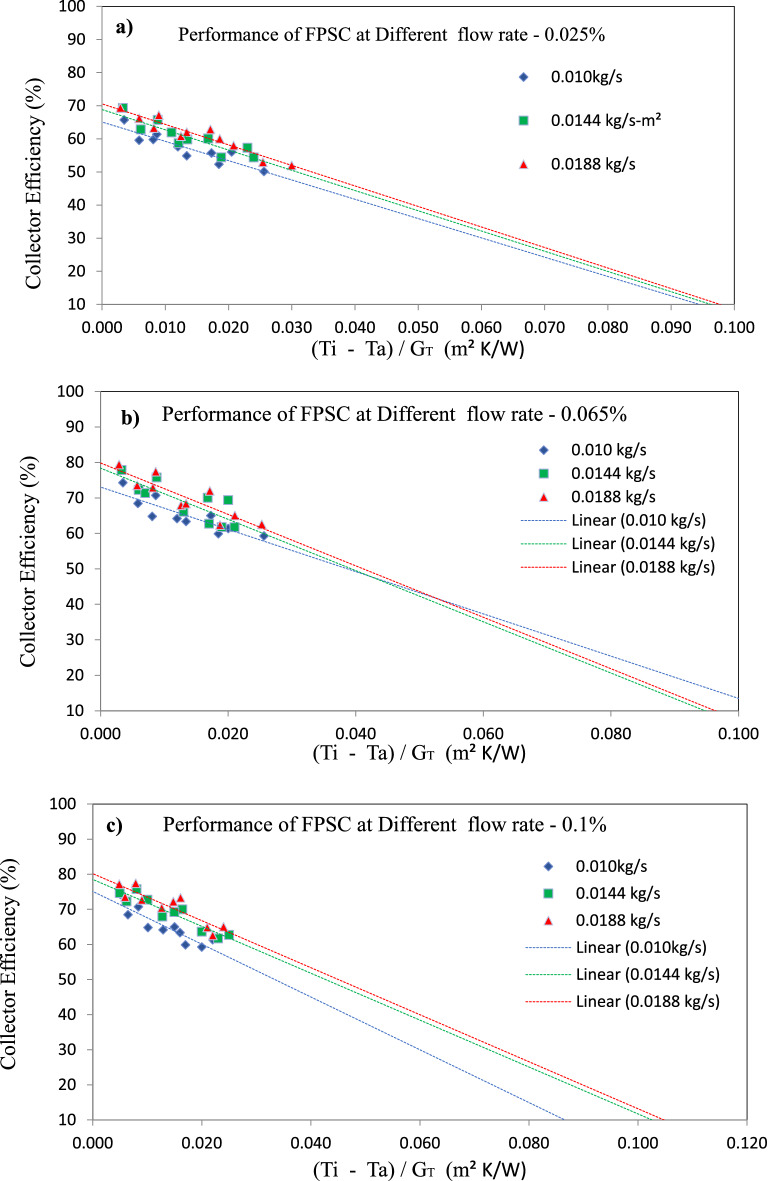
Variation in collector efficiency with $\frac{\left({{\varvec{T}}}_{{\varvec{i}}}-{{\varvec{T}}}_{{\varvec{a}}}\right)}{{{\varvec{G}}}_{{\varvec{T}}}}$ at different weight concentrations (**a**) 0.025%, (**b**) 0.065%, (**c**) 0.1%

Figure 6 presents the relationship between the thermal efficiency of FPSC and the reduced temperature parameter $\frac{\left({T}_{i}-{T}_{a}\right)}{{G}_{T}}$ for various mass flow rates of distilled water as base fluid and GAMWCNTs based nanofluid at different weight fractions of GAMWCNTs nanofluids. It can be noted that GAMWCNTs nanofluids have greater F_R_ (τα) values than base fluid. The highest value was attained at 0.0188 kg/s flow rate and 0.1% wt. concentration. The heat transfer rate is improved with increasing values of heat absorbed factor due to thinner thermal boundary layer thickness.

The heat coefficient for convection (h), whose value is proportional to the thermal conductivity (K) of the fluid utilized, improves the thermal efficiency of FPSC. The substantial improvement in convective coefficient (h) is primarily attributable to developing of a thin thermal boundary layer at the riser tube walls due to the GAMWCNTs nanofluid's increased thermal conductivity and reduction in the thermal resistance between the heat transfer fluid and riser tube inner wall surface. Furthermore, the thickness of the thermal boundary layer is reduced using carbon-based nanoparticles like GNP and MWCNTs. The improved heat transfer coefficient (h) and thermal efficiency of flat plate solar collectors are also attributed to the specific surface area (SSA) and Brownian motion of GAMWCNTs in distilled water.

Compared to base fluid deionized water, there is an increment in energy loss factors for GAMWCNTs nanofluids at various flow rates, as shown in Table 3. Furthermore, the energy absorbed factor values rise with an increase in mass flow rate, as seen in Table 3. It is noted that with increasing GAMWCNT weight fraction compared to deionized water, the augmentation in energy absorbed parameter is 16.99%, 23.70%, and 28.07% at 0.0188 kg/s mass flow rate. The energy loss parameter is 6.17%, 6.69%, and 7.03%.

### Effect of outlet temperature on the thermal performance of the collector

**Figure 7:**
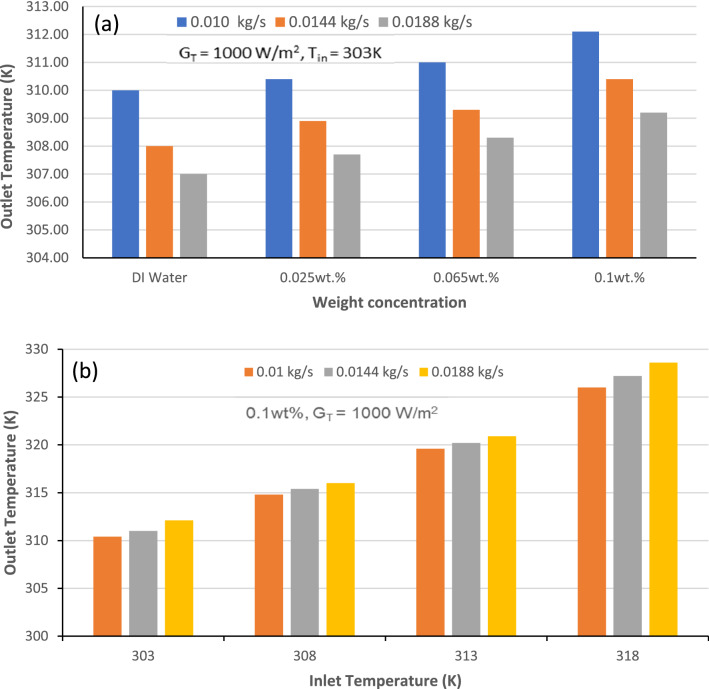
Outlet temperature versus (**a**) weight concentration for different mass flow rates of the working fluid, (**b**) inlet temperature of working fluid.

Many factors affect the efficiency of a flat plate solar collector, and one of the important factor is temperature gradient (ΔT) of the working fluid inside the collector. There is an improvement in thermal performance of FPSC with the temperature gradient because thermal efficiency is directly propotional to the difference of temperature between outlet and inlet as presented in Eq. (2). Moreover, inlet temperature is fixed for specific test run and enhancement in outlet temperature is achieved by utilizing nanofluids in comparison with base fluid.This enhanced value of outlet temperature positively effects thermal efficiency of the FPSC. Figure 7a presents the variation in outlet temperature at different weight fraction for various mass flow rates of GAMWCNT nanofluid at constant G_T_ and inlet temperature. It can be seen that at a particular weight concentration, the temperature at the outlet reduces with the rise in the flow rate of the operating fluid. The deionized water and 0.1% weight concentration of GAMWCNT nanofluid have a 0.8710% and 0.9292% reduction in outlet temperature, respectively. On the other hand, outlet temperature increases with the weight concentration of GAMWCNT nanofluid in the solar collector. Compared to base fluid, the value of outlet temperature was high for various concentrations of GAMWCNTs nanofluid. The enhancement in temperature was 0.6774%, 0.6489% and 0.6183% when base fluid deionized water was replaced by 0.1% weight concentration of GAMWCNT nanofluid as operating fluid at 0.010, 0.0144 and 0.0188 kg/s respectively. There was an improvement in the heat gain value and thermal performance of FPSC due to an increase in the weight concentration of nanofluid. Thus, thermal efficiency is enhanced considerably by utilizing GAMWCNT nanofluid instead of base fluid water. The variation of outlet temperature with inlet temperature by keeping heat flux and weight concentration of operating fluid constant is also investigated, and the results are presented in Fig. 7b. It is observed that an increment in the output temperature occurs when the inlet temperature increases at a specific flow rate. The enhancement in outlet temperature was 4.78% at 0.010 kg/s, 4.95% at 0.0144 kg/s and 5.02% at 0.0188 kg/s in comparison to the inlet temperature. Due to enhancement in outlet temperature, the larger temperature difference is apparent when utilizing GAMWCNT nanofluids compared to deionized water, even though the value of Cp for GAMWCNTs is less than the deionized water (base fluid), leading to the higher thermal performance of the solar collector^55,56^.

### Exergy analysis

**Figure 8:**
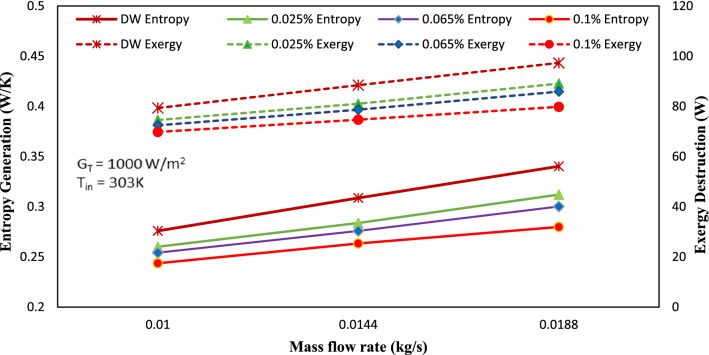
Entropy generation and exergy destruction for base fluid and GAMWCNT nanofluid.

The values of entropy generation (S_gen_)and exergy destruction (E_dest_) significantly impact the exergy efficiency of heat transfer systems. Minimizing E_dest_ and S_gen_ improves energetic performance in these systems. The variation in E_dest_ and S_gen_ values for 0.010, 0.0144, 0.0188 kg/s by keeping heat flux (G_T_) and temperature at the inlet constant is presented in Fig. 8. According to the results, there was an enhancement in the values of entropy generation (S_gen_) and exergy destruction (E_dest_) with the rise in mass flow rate from 0.010 to 0.0188 kg/s for same weight fraction of working fluid. This increment in values of E_dest_ and S_gen_ was due to heat gain increasing as the mass flow rate rises and the outlet temperature of heat transfer fluid falls rapidly. On the other hand, for an increase in GAMWCNT weight fraction at a fixed mass flow rate, there was an enhancement in the value of heat gain factor and outlet temperature with a cost of increased friction factor (Fr). Consequently, the values of exergy destruction and entropy generation are reduced. Due to its superior capacity for heat absorption, 0.1% GAMWCNT nanofluid yields the lowest values of exergy destruction and entropy generation.

**Figure 9:**
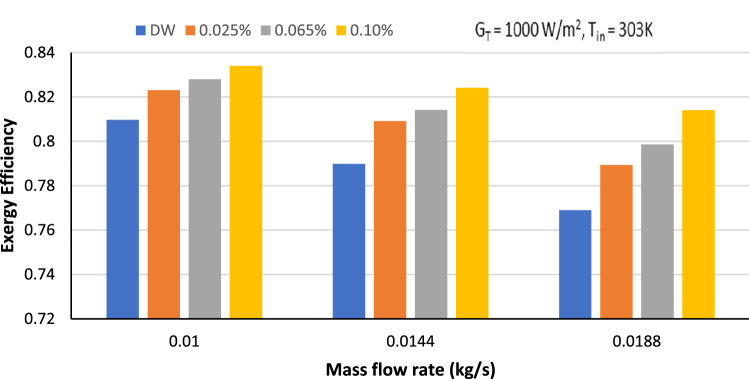
Exergy efficiency versus mass flow rate for base fluid and GAMWCNT nanofluid.

Figure 9 displays the variation in exergy efficiency (η_e_) for GAMWCNT-based nanofluid at 0.010, 0.0144 and 0.0188 kg/s mass flow rate. For a given weight fraction, it has been found that exergy efficiency falls as the flow rate increases. Increasing values of S_gen_ are the main cause of this. Furthermore, the exergy efficiency rises instantaneously with increasing concentration of working fluid at a fixed mass flow rate. Compared to base fluid, higher weight concentrations of GAMWCNTs demonstrated greater exergy efficiency values. At 0.025,0.065 and 0.1% concentration of GAMWCNTs for 0.0188 kg/s, the improvement in exergy efficiency is 2.57%,4.18% and 5.53%, respectively, in comparison to the base fluid. The increment in exergy efficiency is 2.38%, 3.45%, 4.16% at 0.0144 kg/s mass flow rate and 1.62%, 2.42%, 2.91% at 0.010 kg/s for 0.025%, 0.065% and 0.10% weight concentration respectively.

### Friction factor and pumping power

**Figure 10:**
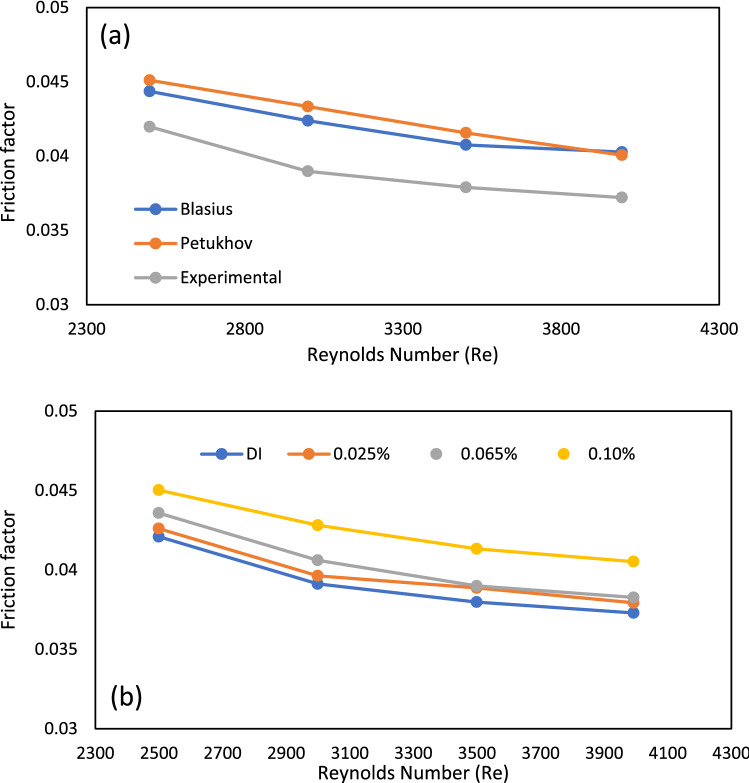
(**a**) Experimental, Blasius and Petukhov friction values of base fluid (DI water) for different Reynold numbers. (**b**) Friction factor values for GAMWCNT nanofluid and DI water at varying Reynold numbers.

Increased values of friction factor and pumping power adversely affect the thermal performance of solar thermal systems, so values of these parameters should be minimum. Figure 10a displays the theoretical friction factor computed from Petukhov and Blasius empirical models and the friction factor determined from experiments on base fluid deionized water at fixed inlet temperature, heat flux and varying Reynold No. (Re). Including some variance, the fair agreement is found between values of these two types of friction factors (theoretical and experimental). It is noticed that the discrepancy between the experimental friction value (f) and the Blasius model is 7.23%, while the difference between the observed friction value and the Petukhov model is 8.26%.

The variation in friction factor values of GAMWCNTs nanofluid at various Reynolds numbers is presented in Fig. 10b. The values obtained for various nanofluid concentrations are compared with base fluid. It is observed that friction factor values decrease with the increase in Reynolds number. This is because when the Reynolds number increases, the density gradient decreases, lowering the magnitude of frictional resistance. On the other hand, as the concentration of GAMWCNTs rises, there is a small increment in friction values compared to deionized water. When GAMWCNTs are dispersed in the base fluid, the nanofluid's viscosity grows, causing pressure drop and, ultimately, friction factor. Compared to base fluid, for 0.025, 0.65 and 0.1% weight fraction of GAMWCNT, the highest rise in friction factor (f) is 2.29, 3.66 and 8.63%. The increased weight concentration of GAMWCNT promotes pressure drop and pumping power because frictional shear forces are induced at greater viscosity and working fluid velocities.

**Figure 11:**
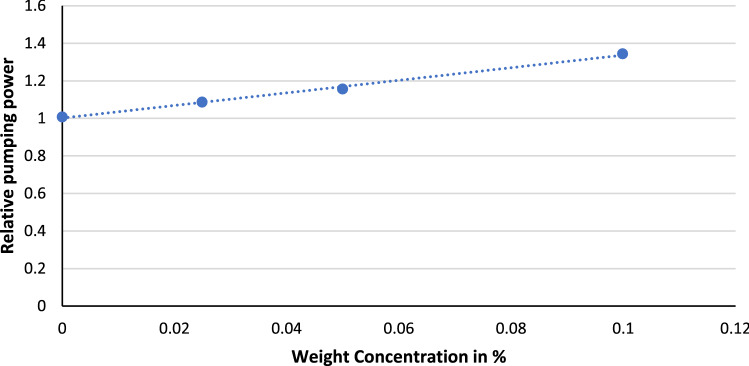
Relative pumping power at varying weight concentrations.

The relative pumping power of GAMWCNTs and base fluid (DW) is shown in Fig. 11. It is observed that there is a slight increase in relative pumping power as nanoparticles' weight concentration increases. However, the pumping power of GAMWCNTs nanofluid and base fluid deionized water is very close.

### Performance index (PI)

**Figure 12:**
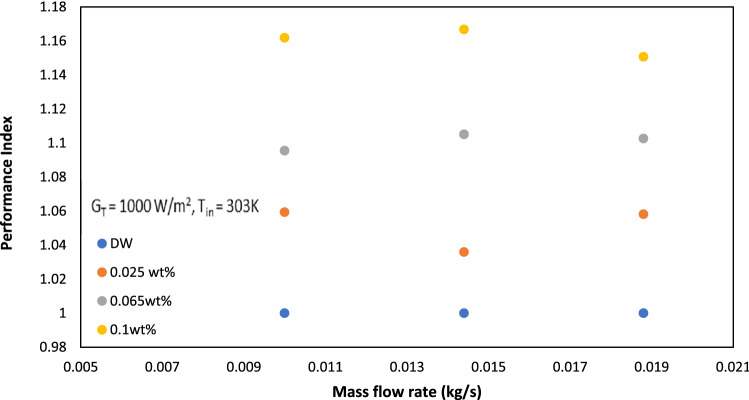
Performance index at various mass flow rates for varying weight concentrations of GAMWCNTs.

Performance index (PI) is a key parameter to assess the effectiveness of GAMWCNT-H_2_O nanofluid in heat transfer systems like flat plate solar collectors. It is essential to remember that nanofluid used in solar collectors must-have performance index values of more than one, as failure to do so will negate any potential benefits and this specific nanofluid is not an acceptable operating fluid^32,46^. Figure 12 displays the performance index values at different flow rates. It is observed that for all weight concentrations of GAMWCNT, performance index parameters of more than one are found because the rise in efficiency of the flat plate collector outweighs the increase in pressure drop value. Furthermore, the values of PI increase with the rise in the weight concentration of GAMWCNT. Hence, higher concentration GAMWCNT nanofluid with increased Performance index and efficiency can be a viable alternative operating fluid in FPSC.

### Size reduction of flat plate solar collector

**Figure 13:**
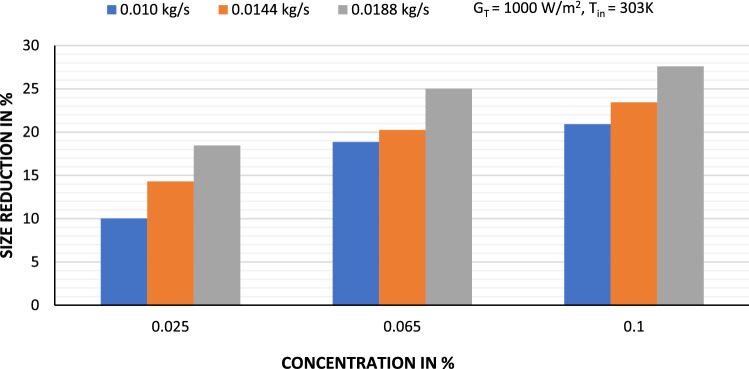
Size reduction of FPSC at different weight concentrations of GAMWCNT nanofluid.

The primary objective of this investigation is to evaluate how much energy and material may be saved in the development of FPSC with GAMWCNT nanofluids as heat transfer fluids. Figure 13 shows the possible size reduction at a different weight concentration of GAMWCNT nanofluid in a flat plate collector. It has been found that there is an enhancement in size reduction of the collector with the rise in flow rate at the fixed concentration of GAMWCNT nanofluid. Moreover, at a constant flow rate, increasing GAMWCNT concentration enhanced the possibility for flat plate solar collector size reduction. It is recorded that when FPSC operated at 0.0188 kg/s and 0.1% GAMWCNT nanofluid concentration, the highest size reduction, 27.59%, was attained as compared to FPSC with water as heat transfer fluid. Thus, FPSC using GAMWCNT nanofluid is more cost-effective than FPSC using water.

### Economic analysis

The computation of all the energy required to construct a product or object is known as embodied energy. The ongoing advancement of industrial technology is due to decreased embodied energy. Various studies demonstrate that using nanofluids reduces energy production costs compared to using water. Since more useful energy is produced using nanofluids, the collector's energy production costs are reduced, and its thermal performance is improved^57–59^. Economic analysis heavily relies on assessing embodied energy in flat plate solar collectors. Effective evaluation of the economic implications of flat plate collectors was done using the life cycle assessment approach^34,47,60,61^. Because more than 70% of EE originated from the construction of FPSC, the methodology adopted only considers the embodied energy (EE) during the construction and operation phases of FPSC^62,63^. The present research considers how mass and embodied energy affect the lowering of the flat plate collector size. At various concentration of GAMWCNT nanofluid and base fluid, the economics and embodied energy analysis is presented in Table 4. Glass and copper are the two main components of the solar collector. The embodied energy indexes for glass and copper are 15.9 MJ/kg and 70.6 MJ/kg, respectively^64,65^. The present analysis considers the size reduction of FPSC as a function of mass and embodied energy. It was found that the size of FPSC was decreased when GAMWCNT nanofluid was used in place of base fluid water, saving 321.72 MJ of embodied energy.

**Table 4:** Economic analysis of FPSC with water and GAMWCNT–water nanofluid.

	FPSC (Water)	FPSC (0.025% GAMWCNT)	FPSC (0.065% GAMWCNT)	FPSC (0.1% GAMWCNT)
EE of FPSC (MJ)	1167.1	951.79	875.2	845.38
Energy saving in %		18.448	25.011	27.595
Collector cost (USD)	252.4	205.83	189.27	182.75
Entire system cost (USD)	770.94	770.94	770.94	770.94
Cost of GAMWCNT nanofluid		1.43	3.718	5.72
System total cost (USD)	1023.34	978.2	963.92	959.41
Cost saving in the form of electricity per annum (USD)	505.75	505.75	505.75	505.75
Years until electricity saving	2.023	1.934	1.905	1.897

Additionally, as the area of the flat plate collector is reduced, there is a decrease in the demand for electricity, which lowers system operating costs. The payback period was 1.897 years for FPSC with GAMWCNT nanofluids at 0.1 wt.%, which was 6.228% shorter than using water as a heat transfer fluid. Therefore, it is concluded that FP solar collector with GAMWCNT nanofluid as heat transfer fluid is more efficient and saves more energy than FPSC with water.

## Conclusion

Experimental research was done to analyze the effects of green synthesized GAMWCNT nanofluid, a non-corrosive, non-toxic, and environmentally friendly heat transfer fluid, on the performance of flat plate solar collectors.

The followings are the important points of the conclusion.

- The stability analysis test showed higher stability of GAMWCNTs in base fluid for 60 days without aggregation.
- The collector's thermal efficiency was increased with the rise in heat flux, mass flow rate and weight concentration, but a decline was seen as the inlet temperature went up. As per experimental findings, the highest improvement in energy efficiency was 30.8% for a 0.1% weight concentration of GAMWCNT nanofluid at 0.0188 kg/s compared to the base fluid.
- Exergy analysis revealed that exergy efficiency (${\eta }_{e})$ increases with the enhancement in GAMWCNT weight concentration but decreases with the increment in flow rate. The maximum exergy efficiency was attained at 0.1% GAMWCNT concentration and 0.010 kg/s mass flow rate.
- For GAMWCNT nanofluid concentrations of 0.025, 0.065, and 0.1% compared to base fluid (DW), the maximum rise in friction factor was nearly 2.29, 3.66 and 8.63%.
- Performance index (PI) values of more than 1 were achieved for each weight concentration of GAMWCNT-H_2_O nanofluid. A rise in GAMWCNT nanofluid concentration showed higher values for PI. The payback period was 1.897 years for FPSC with GAMWCNT nanofluids which were 6.228% shorter than using water as heat transfer fluid. It is therefore concluded that FP solar collectors with GAMWCNT nanofluid as the heat transfer fluid are more effective and save more energy than FPSCs with water.

## Future scope of the study

Depending on the findings of the current study, the following specific aspects may be taken into account in subsequent studies on FPSCs based on nanofluids:

- The preparation of nanomaterials with a greater specific surface area will require special attention from researchers to ensure their excellent colloidal stability, thermophysical characteristics, and thermal performance of the FPSCs.
- Nanofluids must be stable in colloidal suspensions to be chosen as heat transfer fluids. Poorly prepared nanofluids have a propensity to clump together and settle, which could clog the flow channels and lower their thermal conductivity. Therefore, for successful usage in FSPCs or other heat transfer systems, researchers must focus on synthesising nanofluids with long colloidal stability in suspension.

## Data Availability

The datasets used and analyzed during the current study are available from the corresponding author upon reasonable request.

## List of symbols

A_c_: Area of flat plate collector (m^2^)
A_RC_: Reduction in the area of the collector (m^2^)
NIST: National Institute of Standards and Technology
*C*_p_: Heat capacity of fluid (J/kg K)
CNTs: Carbon nanotubes
MWCNT: Multi-walled carbon nanotube
GAMWCNT: Gallic Acid based multi-walled carbon nanotube
GGNP: Gallic acid-treated GNP
FPSC: Flat plate solar collector
TEM: Transmission Electron Microscopy
GA: Gallic Acid
GNPs: Graphene nanoplatelets
H_2_O_2_: Hydrogen peroxide
*wt%*: Weight percentage/mass fraction
*K*: Thermal conductivity of the fluid (W/m.K)
K: Kelvin
L: Length of the tube (m)
$\dot{m}$: Working fluid flow rate (kg/s)
UV–VIS: Ultraviolet–visible spectroscopy
D_i_: Inner diameter
$${\dot{\text{q}}}$$: Heat flux (W/m^2^)
bf: Base fluid
nf: Nanofluid
h: Convective heat transfer coefficient (W/m^2^. K)
Re: Reynolds number
ƒ: Experimental friction factor
T: Temperature, °C
TiO_2_: Titanium dioxide
CeO_2_: Cerium (IV) oxide
Al_2_O_3_: Aluminum oxide
ZnO: Zinc oxide
RTD: Resistance temperature detector
FTIR: Fourier transform infrared spectroscopy
TEA: Triethanolamine
ASHRAE: American Society of Heating, Refrigeration, and Air- Conditioning Engineers
SSA: Specific surface area
G_T_: The intensity of heat flux (W/m^2^)
F_R_ (τα): Heat absorbed factor
F_R_U_L_: Heat removal factor
E_dest_: Exergy destruction
S_gen_: Entropy generation
PI: Performance index
EE: Embodied energy
TW: Tube wall
*T*_*a*_: Ambient temperature (K)
*T*_*i*_: The fluid temperature at the inlet(K)
*T*_*o*_: The fluid temperature at the outlet (K)
*T*_*s*_: The temperature of surroundings (K)

## Greek symbol

$\rho$: Fluid density(kg/m^3^)
Δp: Difference of pressure (Pa)
*η*_*e*_: Exergy efficiency
*η*: Collector thermal efficiency
µ: Fluid dynamic viscosity (mPa s)

## References

[1] Agency, I. A. E. *World Energy Outlook, Organization For Economic, S.I* (2016).

[2] London, B. *BP Statistical Review of World Energy June 2016* (2016).

[3] Bellos E, Tzivanidis CJTS, Progress E (2017). Parametric investigation of nanofluids utilization in parabolic trough collectors. Therm. Sci. Eng. Progress.

[4] Diego-Ayala U, Carrillo JJRE (2016). Evaluation of temperature and efficiency in relation to mass flow on a solar flat plate collector in Mexico. Renew. Energy.

[5] Kalogirou SA, Karellas S, Badescu V, Braimakis KJRE (2016). Exergy analysis on solar thermal systems: A better understanding of their sustainability. Renew. Energy.

[6] Mahian O (2018). Recent advances in modeling and simulation of nanofluid flows-part I: Fundamental and theory. Phys. Rep..

[7] Sakhaei SA, Valipour MSJJOTA, Calorimetry (2020). Investigation on the effect of different coated absorber plates on the thermal efficiency of the flat-plate solar collector. J. Therm. Anal. Calorim..

[8] Mahian O (2018). Recent advances in modeling and simulation of nanofluid flows-part II: applications. Phys. Rep..

[9] Amber K (2018). Heating and cooling degree-days maps of Pakistan. Energies.

[10] Bellos E, Tzivanidis CJJOTA, Calorimetry (2018). A review of concentrating solar thermal collectors with and without nanofluids. J. Therm. Anal. Calorim..

[11] Deeyoko LAJ, Balaji K, Iniyan S, Sharmeela CJATE (2019). Exergy, economics and pumping power analyses of flat plate solar water heater using thermal performance enhancer in absorber tube. Appl. Therm. Eng..

[12] Raj P, Subudhi SJR, Reviews SE (2018). A review of studies using nanofluids in flat-plate and direct absorption solar collectors. Renew. Sustain. Energy Rev..

[13] Choi, S. U. & Eastman, J. A. *Enhancing Thermal Conductivity of Fluids with Nanoparticles* (Argonne National Lab, 1995).

[14] Lee, S., Choi, S.-S., Li, S. & Eastman, J. *Measuring Thermal Conductivity of Fluids Containing Oxide Nanoparticles* (1999).

[15] Masuda AEH, Teramae K (1993). Alteration of thermal conductivity and viscosity of liquid by dispersing ultra-fine particles. Dispersion of Al2O3, SiO2 and TiO2 ultra-fine particles. Netsu Bussei.

[16] Yousefi T, Shojaeizadeh E, Veysi F, Zinadini S (2012). An experimental investigation on the effect of pH variation of MWCNT–H2O nanofluid on the efficiency of a flat-plate solar collector. Sol. Energy.

[17] Said Z (2015). Performance enhancement of a flat plate solar collector using titanium dioxide nanofluid and polyethylene glycol dispersant, (in English). J. Clean. Prod..

[18] He Q, Zeng S, Wang S (2015). Experimental investigation on the efficiency of flat-plate solar collectors with nanofluids. Appl. Therm. Eng..

[19] Hajabdollahi H, Khosravian M, Dehaj MSJE (2022). Thermo-economic modeling and optimization of a solar network using flat plate collectors. Energy.

[20] Said Z, Sharma P, Tiwari AK, Huang Z, Bui VG, Hoang ATJJOCP (2022). Application of novel framework based on ensemble boosted regression trees and Gaussian process regression in modelling thermal performance of small-scale organic rankine cycle using hybrid nanofluid. J. Clean. Prod..

[21] Ahmadi A, Ganji DD, Jafarkazemi F (2016). Analysis of utilizing Graphene nanoplatelets to enhance thermal performance of flat plate solar collectors. Energy Convers. Manage..

[22] Said Z, Sharma P, Aslfattahi N, Ghodbane MJJOES (2022). Experimental analysis of novel ionic liquid-MXene hybrid nanofluid's energy storage properties: Model-prediction using modern ensemble machine learning methods. J. Energy Storage.

[23] Mustafa J, Alqaed S, Sharifpur MJSET, Assessments (2022). Evaluation of energy efficiency, visualized energy, and production of environmental pollutants of a solar flat plate collector containing hybrid nanofluid. Sustain. Energy Technol. Assess..

[24] Said Z, Rahman S, Sharma P, Hachicha AA, Issa SJATE (2022). Performance characterization of a solar-powered shell and tube heat exchanger utilizing MWCNTs/Water-based nanofluids: An experimental, Numerical, and Artificial Intelligence approach. Appl. Therm. Eng..

[25] Jouybari HJ, Saedodin S, Zamzamian A, Nimvari ME, Wongwises S (2017). Effects of porous material and nanoparticles on the thermal performance of a flat plate solar collector: An experimental study. Renew. Energy.

[26] Kilic F, Menlik T, Sozen A (2018). Effect of titanium dioxide/water nanofluid use on thermal performance of the flat plate solar collector, (in English). Sol. Energy.

[27] Mondragon R, Sanchez D, Cabello R, Llopis R, Julia JE (2019). Flat plate solar collector performance using alumina nanofluids: Experimental characterization and efficiency tests, (in English). PLoS ONE.

[28] Arora S, Fekadu G, Subudhi SJJOSEE (2019). Energy and exergy analysis of marquise shaped channel flat plate solar collector using Al2O3–water nanofluid and water. J. Solar Energy Eng..

[29] Akram N (2019). An experimental investigation on the performance of a flat-plate solar collector using eco-friendly treated graphene nanoplatelets–water nanofluids. J. Therm. Anal. Calorim..

[30] Choudhary S, Sachdeva A, Kumar PJRE (2020). Influence of stable zinc oxide nanofluid on thermal characteristics of flat plate solar collector. Renew. Energy.

[31] Moravej M (2020). Enhancing the efficiency of a symmetric flat-plate solar collector via the use of rutile TiO2-water nanofluids. Sustain. Energy Technol. Assess..

[32] Sarsam WS, Kazi SN, Badarudin AJATE (2020). Thermal performance of a flat-plate solar collector using aqueous colloidal dispersions of graphene nanoplatelets with different specific surface areas. Appl. Therm. Eng..

[33] Akram N (2021). Experimental investigations of the performance of a flat-plate solar collector using carbon and metal oxides based nanofluids. Energy.

[34] Kumar LH, Kazi S, Masjuki H, Zubir M, Jahan A, Bhinitha CJATE (2021). Energy, exergy and economic analysis of liquid flat-plate solar collector using green covalent functionalized graphene nanoplatelets. Appl. Therm. Eng..

[35] Sadri R (2018). A facile, bio-based, novel approach for synthesis of covalently functionalized graphene nanoplatelet nano-coolants toward improved thermo-physical and heat transfer properties. J. Colloid Interface Sci..

[36] Sadri R (2017). A novel, eco-friendly technique for covalent functionalization of graphene nanoplatelets and the potential of their nanofluids for heat transfer applications. Chem. Phys. Lett..

[37] Golumbic C, Mattill HJO, Soap (1942). The antioxidant properties of gallic acid and allied compounds. Oil Soap.

[38] Akram N (2022). A facile, green fabrication of aqueous nanofluids containing hydrophilic functionalized carbon nanotubes toward improving heat transfer in a closed horizontal flow passage. Powder Technol..

[39] A. Standard, Standard 93-2003. *Method of Testing to Determine the Thermal** Performance of Solar Collector* (2003).

[40] Tong Y, Chi X, Kang W, Cho HJATE (2020). Comparative investigation of efficiency sensitivity in a flat plate solar collector according to nanofluids. Appl. Therm. Eng..

[41] Verma SK, Tiwari AK, Chauhan DS (2017). Experimental evaluation of flat plate solar collector using nanofluids. Energy Convers. Manage..

[42] Vincely DA, Natarajan E (2016). Experimental investigation of the solar FPC performance using graphene oxide nanofluid under forced circulation, (in English). Energy Convers. Manage..

[43] Petukhov BJNY (1970). Advances in Heat Transfer.

[44] Blasius H (1907). Grenzschichten in Flüssigkeiten mit kleiner Reibung.

[45] Sadri R (2017). Study of environmentally friendly and facile functionalization of graphene nanoplatelet and its application in convective heat transfer. Energy Convers. Manage..

[46] Razi P, Akhavan-Behabadi M, Saeedinia MJICIH, Transfer M (2011). Pressure drop and thermal characteristics of CuO–base oil nanofluid laminar flow in flattened tubes under constant heat flux. Int. Commun. Heat Mass Transfer.

[47] Michael-Joseph-Stalin P, Arjunan T, Matheswaran M, Dolli H, Sadanandam NJJOTA, Calorimetry (2020). Energy, economic and environmental investigation of a flat plate solar collector with CeO2/water nanofluid. J. Therm. Anal. Calorim..

[48] Faizal M, Saidur R, Mekhilef S, Alim MA (2013). Energy, economic and environmental analysis of metal oxides nanofluid for flat-plate solar collector, (in English). Energy Convers. Manage..

[49] Kline SJJME (1953). Describing uncertainty in single sample experiments. Mech. Engineering.

[50] Ramires ML (1995). Standard reference data for the thermal conductivity of water. J. Phys. Chem. Ref. Data.

[51] Aravind SJ, Baskar P, Baby TT, Sabareesh RK, Das S, Ramaprabhu SJTJOPCC (2011). Investigation of structural stability, dispersion, viscosity, and conductive heat transfer properties of functionalized carbon nanotube based nanofluids. J. Phys. Chem. C.

[52] Goodarzi M, Toghraie D, Reiszadeh M, Afrand MJJOTA, Calorimetry (2019). Experimental evaluation of dynamic viscosity of ZnO–MWCNTs/engine oil hybrid nanolubricant based on changes in temperature and concentration. J. Therm. Anal. Calorim..

[53] Goodarzi M (2015). Investigation of heat transfer and pressure drop of a counter flow corrugated plate heat exchanger using MWCNT based nanofluids. Int. Commun. Heat Mass Transfer.

[54] Park JJ, Park DM, Youk JH, Yu W-R, Lee JJC (2010). Functionalization of multi-walled carbon nanotubes by free radical graft polymerization initiated from photoinduced surface groups. Carbon.

[55] Alawi OA, Kamar HM, Mallah A, Kazi S, Sidik NACJSE (2019). Thermal efficiency of a flat-plate solar collector filled with pentaethylene glycol-treated graphene nanoplatelets: An experimental analysis. Sol. Energy.

[56] Ahmadi A, Ganji DD, Jafarkazemi FJEC, Management (2016). Analysis of utilizing Graphene nanoplatelets to enhance thermal performance of flat plate solar collectors. Energy Convers. Manage..

[57] Saray JA, Heyhat MMJIJOER (2022). Multi-objective assessment of a DAPTC based on 4E analysis: Water-energy-environment nexus. Int. J. Energy Res..

[58] Mashhadian A, Heyhat MMJES (2022). Part A: Recovery, utilization,, and E. Effects, Energy, exergy, and environmental assessments of a direct absorption parabolic trough collector based on nanofluid volume absorption approach. Energy Sourc..

[59] Saray JA, Heyhat MMJE (2022). Modeling of a direct absorption parabolic trough collector based on using nanofluid: 4E assessment and water-energy nexus analysis. Energy.

[60] Faizal M, Saidur R, Mekhilef S, Hepbasli A, Mahbubul IM (2015). Energy, economic, and environmental analysis of a flat-plate solar collector operated with SiO2 nanofluid, (in English). Clean Technol. Environ. Policy.

[61] Kalogirou SJSE (2009). Thermal performance, economic and environmental life cycle analysis of thermosiphon solar water heaters. Sol. Energy.

[62] Ardente F, Beccali G, Cellura M, Brano VLJRE (2005). Life cycle assessment of a solar thermal collector. Renew. Energy.

[63] Mashhadian A, Heyhat MM, Mahian OJEC, Management (2021). "Improving environmental performance of a direct absorption parabolic trough collector by using hybrid nanofluids. Energy Convers. Manage..

[64] Otanicar TP, Phelan PE, Prasher RS, Rosengarten G, Taylor RAJJor, s. energy (2010). "Nanofluid-based direct absorption solar collector. J. Renew. Sustain. Energy.

[65] Faizal M, Saidur R, Mekhilef S, Hepbasli A, Mahbubul IJCT, Policy E (2015). Energy, economic, and environmental analysis of a flat-plate solar collector operated with SiO2 nanofluid. Clean Technol. Environ. Policy.

